# Premature aging in telomerase-deficient zebrafish

**DOI:** 10.1242/dmm.011635

**Published:** 2013-06-05

**Authors:** Monique Anchelin, Francisca Alcaraz-Pérez, Carlos M. Martínez, Manuel Bernabé-García, Victoriano Mulero, María L. Cayuela

**Affiliations:** 1 Telomerase, Aging and Cancer Group, Research Unit, Department of Surgery, CIBERehd, University Hospital “Virgen de la Arrixaca”, Murcia, Spain; 2 Instituto Murciano de Investigación Biosanitaria (IMIB), Murcia, Spain; 3 Departamento de Biología Celular e Histología, Facultad de Biología, Universidad de Murcia, Murcia, Spain

## Abstract

The study of telomere biology is crucial to the understanding of aging and cancer. In the pursuit of greater knowledge in the field of human telomere biology, the mouse has been used extensively as a model. However, there are fundamental differences between mouse and human cells. Therefore, additional models are required. In light of this, we have characterized telomerase-deficient zebrafish (*Danio rerio*) as the second vertebrate model for human telomerase-driven diseases. We found that telomerase-deficient zebrafish show p53-dependent premature aging and reduced lifespan in the first generation, as occurs in humans but not in mice, probably reflecting the similar telomere length in fish and humans. Among these aging symptoms, spinal curvature, liver and retina degeneration, and infertility were the most remarkable. Although the second-generation embryos died in early developmental stages, restoration of telomerase activity rescued telomere length and survival, indicating that telomerase dosage is crucial. Importantly, this model also reproduces the disease anticipation observed in humans with dyskeratosis congenita (DC). Thus, telomerase haploinsufficiency leads to anticipation phenomenon in longevity, which is related to telomere shortening and, specifically, with the proportion of short telomeres. Furthermore, p53 was induced by telomere attrition, leading to growth arrest and apoptosis. Importantly, genetic inhibition of p53 rescued the adverse effects of telomere loss, indicating that the molecular mechanisms induced by telomere shortening are conserved from fish to mammals. The partial rescue of telomere length and longevity by restoration of telomerase activity, together with the feasibility of the zebrafish for high-throughput chemical screening, both point to the usefulness of this model for the discovery of new drugs able to reactivate telomerase in individuals with DC.

## INTRODUCTION

In most organisms, telomeres are composed of simple repetitive sequences whose length is maintained by the telomerase ribonucleoprotein. Telomerase contains two essential components: the telomerase reverse transcriptase (TERT) and telomerase RNA (TR), which provides the template for the reverse transcription of new telomere DNA by TERT (Blackburn, 1991). Loss of telomerase function results in progressive telomere shortening and chromosomal instability, which ends up having implications for aging and cancer (Blasco et al., 1997). Therefore, the study of telomere biology is crucial to the understanding of these two processes (Blasco, 2005).

The mouse has been used extensively as a model for human telomere biology (Goytisolo and Blasco, 2002). However, there are strong differences between mouse and human telomere length (Autexier, 2008). Therefore, other models have been characterized to clarify the role played by telomerase in aging and cancer, such as *Saccharomyces cerevisiae* (Cohn and Blackburn, 1995), *Caenorhabditis elegans* (Wicky et al., 1996), *Arabidopsis thaliana* (Fitzgerald et al., 1999), *Gallus gallus* (Swanberg et al., 2010) and *Danio rerio* (Anchelin et al., 2011). The zebrafish (*Danio rerio*) has the potential to emerge as a key vertebrate model in this regard. In fact, during the last decade, several zebrafish studies about the role of the TERT complex in aging, cancer and regeneration have been reported (Anchelin et al., 2011; Imamura et al., 2008). The zebrafish TERT and TR components have been cloned and characterized (Lau et al., 2008). In terms of telomere length, zebrafish telomeres (15–20 kb) are relatively similar to human telomeres (10–15 kb). Although telomerase is constitutively active in multiple organs in zebrafish, unlike the situation in the corresponding mammalian tissues, the expression of *tert* mRNA, telomerase activity and telomere length all decreased drastically in almost all of the fish tissues after 18 months of age that we examined, indicating that they are useful markers for evaluating the aging process in zebrafish (Anchelin et al., 2011; Xie et al., 2008).

The use of morpholino-based gene knockdown in zebrafish allows for the modeling of the role of telomerase and telomeres in premature aging syndromes, such as dyskeratosis congenita (DC) (Imamura et al., 2008; Zhang et al., 2012), a human disease characterized by shortened telomeres (Calado and Young, 2009; Kirwan and Dokal, 2009). Thus, TERT or dyskerin knockdown results in embryonic hematopoietic defects at the onset of circulation characterized by the impaired differentiation of blood cells (Imamura et al., 2008) and their eventual apoptosis (Zhang et al., 2012), as occurs in human DC and hypochromic anemia. Furthermore, a number of zebrafish mutants have now been developed as models for human telomeric diseases. For example, the mutation in Nap10, one of the known H/AXA RNP complex genes with mutations linked to DC (Pereboom et al., 2011), or the mutation in the telomeric repeat binding factor 2 (TRF2) (Kishi et al., 2008) have both shown premature aging phenotypes. Therefore, the zebrafish is a powerful model that offers a unique opportunity to contribute to the advancement in biological and behavioral gerontology. The availability of mutant genotypes with identified aging phenotypes, in combination with a wealth of information about zebrafish development and genetics as well as the existence of multiple mutant and transgenic lines, should substantially facilitate the use of this outstanding vertebrate model in deciphering the mechanisms of aging, and in developing preventive and therapeutic strategies to prolong the productive lifespan (‘healthspan’) in humans.

**TRANSLATIONAL IMPACT**

**Clinical issue**

Dyskeratosis congenita (DC) is a rare congenital disorder that is characterized by premature aging and, at the molecular level, the inheritance of short telomeres. The disease is caused by mutations in a number of genes, all of which encode products involved in telomere maintenance. Progressive telomere shortening is a hallmark of many diseases and is also associated with the normal process of aging. Most of our knowledge regarding the role of telomeres and telomerase (the protein that maintains telomere length) in aging has been gained by the analysis of human DC patients and mouse models. Although mice have provided valuable insights into telomere biology, a fundamental difference is that telomere length in mice is longer than in humans, which could explain why mouse models do not recapitulate all the symptoms of DC. Studies in vertebrate models that more closely resemble mammals in terms of telomere length are required to fully understand the implications of telomerase dysfunction in DC and related diseases.

**Results**

Zebrafish, whose telomeres are of a similar length to human telomeres, has recently emerged as an excellent model for high-throughput chemical and genetic screening. In the present study, a telomerase-deficient zebrafish was characterized. The authors report that this model shows signs of premature aging, including spinal curvature, infertility and reduced lifespan in the first generation. Furthermore, the zebrafish mutants display activation of p53 in response to telomere attrition, and show the anticipation phenomenon (the onset of disease at a younger age in the next generation) due to telomerase haploinsufficiency. Crucially, reintroduction of telomerase is able to rescue telomere shortening and longevity in the zebrafish model.

**Implications and future directions**

Overall, telomerase-deficient zebrafish demonstrate several features associated with premature aging in humans. As in mammals, telomerase haploinsufficiency in zebrafish results in anticipation, providing further evidence that telomerase dosage is essential. Unlike equivalent mouse models, the premature aging phenotypes are discernible in the first generation. Thus, these zebrafish provide a model system that overrides the limitations of mice for aging studies, namely the need to breed for several generations (which can be expensive and time-consuming) to obtain the desired phenotype. These characteristics together with the feasibility of zebrafish for high-throughput chemical screening underlines the usefulness of this animal model to clarify the role of telomeres and telomerase in premature aging syndromes such as DC, and for the identification of new therapeutic drugs.

We have thus initiated the characterization of the first zebrafish model of the *tert* gene. Using an identified and publicly available mutant line that carries a mutation in the telomerase gene that was generated by ENU mutagenesis at the Sanger Institute Zebrafish Mutant Project (termed hu3430), we have defined the behavioral, morphological, functional and histopathological features of *tert* mutant zebrafish. The first generation of mutant animals showed patent reduction of telomere length, premature aging, decreased fertility and a shorter lifespan than wild-type zebrafish. These results suggest that telomerase function is crucial for organ homeostasis in zebrafish – as occurs in the mouse (Blasco et al., 1997; Chiang et al., 2004; Yuan et al., 1999) – but only in the first generation. In fact, these mutants can only be bred for one generation owing to their reduced fitness levels. The second generation showed a high percentage of abnormal phenotypes and embryonic lethality, which correlated with drastic telomere shortening and genomic instability. Notably, telomere attrition triggered p53-dependent apoptosis in early embryonic stages and p53 deficiency rescued the adverse effects of telomere loss, as reported in the mouse (González-Suárez et al., 2000; Leri et al., 2003). Therefore, this zebrafish model provides a new platform for examining currently unknown TERT functions in the aging process and discovering drugs for the treatment of premature human aging disorders (Zon and Peterson, 2005).

## RESULTS

### Telomerase deficiency results in both a lack of telomerase activity and telomere shortening

**Fig. 1.**
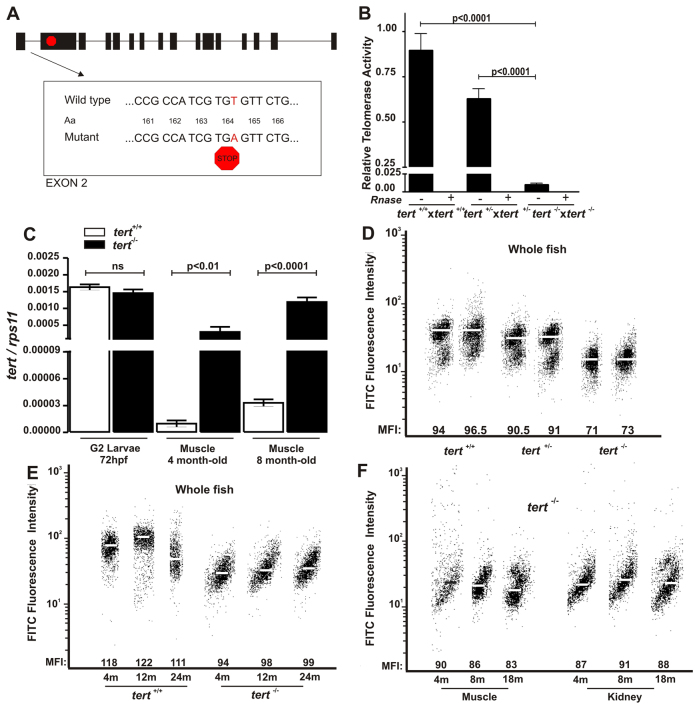
**The dynamics of telomere and telomerase in *tert* mutant zebrafish.** (A) Schematic representation of the telomerase gene mutation. (B) Telomerase activity was measured quantitatively in whole zebrafish embryos (3 dpf, *n*=100) by Q-TRAP using 0.1 mg of protein extract. Results are expressed as the mean value ± s.e.m. from triplicate samples relative to telomerase-positive cells. Statistical significance was assessed using Student’s *t*-test (*P*<0.05). To confirm the specificity of the assay, a negative control is included for each sample, treated with 1 μg of RNase at 37°C for 20 minutes. (C) The mRNA levels of *tert* gene were determined by real-time RT-PCR in larvae and adult muscle tissue of the indicated genotypes. Gene expression is normalized against *rps11*. Each bar represents the mean ± s.e.m. from 100 pooled animals for larvae and three individual fish for adult tissue and triplicate samples. (D) Representation of 3-month-old zebrafish cell distribution according to telomere length. Medium fluorescence intensity (MFI) is indicated for each genetic background. The same trend was observed in the three independent experiments. (E) Representation of wild-type and *tert* mutant zebrafish cell distribution throughout their life according to their telomere length. MFI is indicated for each genetic background. The same trend was observed in the three independent experiments. m, months. (F) Representation of zebrafish muscle and kidney cell distribution throughout life according to their telomere length. MFI is indicated for each genetic background. The same trend was observed in the three independent experiments.

We obtained a publicly available mutant line in the *tert* gene generated by ENU mutagenesis at the Sanger Institute (hu3430). This mutant line has a nonsense mutation (T>A) that results in a premature stop codon and a truncated protein with only the first 156 of the 1088 amino acids and an absence of the RNA-binding and reverse transcriptase domains (Fig. 1A). As expected, this line did not have any telomerase activity (Fig. 1B). Although *tert* gene expression was not affected in larvae, the lack of telomerase activity might have been responsible for the induction of *tert* expression in adult tissues, such as the skeletal muscle (Fig. 1C). In addition, a shorter telomere length was observed in 3-month-old *tert^−/−^* juveniles (Fig. 1D), as assayed by flow-FISH.

Because telomere length has been proposed as a good aging biomarker in the zebrafish model (Anchelin et al., 2011), we measured the mean telomere length of cells from whole mutant fish and several of their organs at three different stages, namely young adult (4-months old), adult (8- or 12-months old) and older adult (18- or 24-months old), in order to determine the relationship between the aging process and telomere length in the absence of telomerase activity. The results show that telomere length of wild-type zebrafish increased between juvenile and the young-adult stage followed by a decrease at the older-adult stage (Fig. 1E), as already reported (Anchelin et al., 2011). In contrast, *tert*^−/−^ specimens showed a telomeric length much shorter than their wild-type siblings but, surprisingly, they were able to maintain telomere length throughout their life (Fig. 1E). We further confirmed this observation in specific tissues, such as those of the muscle and the kidney. Thus, the telomere length of muscle cells slightly decreased throughout the zebrafish lifespan, whereas kidney cells maintained their telomere length (Fig. 1F), indicating that the reduction or maintenance of telomere length is tissue specific in the context of telomerase deficiency in zebrafish (Lee et al., 1998).

### Telomerase deficiency results in a reduced lifespan and premature aging

**Fig. 2.**
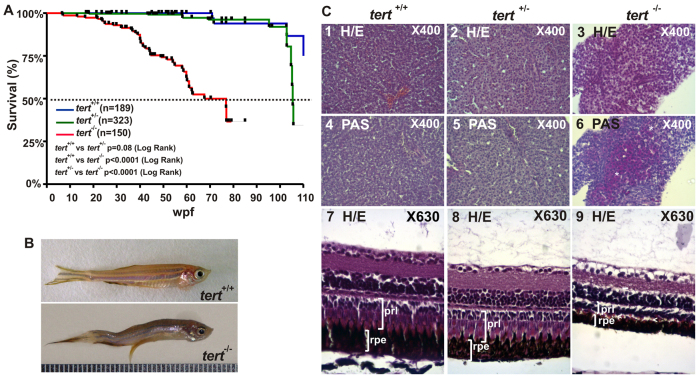
**Premature aging signals in *tert* mutant zebrafish.** (A) Kaplan-Meier representation of the survival of three genetic backgrounds. Post-hatching time is shown in weeks. A dashed line indicates 50% survival. The Log Rank test was used for statistical analysis. (B) Representative image of a *tert^+/+^* and a *tert^−/−^* zebrafish at 11 months old. (C) Liver sections stained with H&E showed cytoplasmic vacuolization of hepatocytes (3) and were PAS-positive (6, white asterisks). H&E-stained eye sections revealed a decreased thickness of both retinal pigmented epithelium (rpe) and the photoreceptor layer (prl), which had a disorganized appearance (9, white arrowheads). Representative images are from at least three fish per genotype.

Outbred zebrafish have a mean lifespan of 42 months and, similar to humans, exhibit a gradual senescence (Gerhard and Cheng, 2002; Gerhard et al., 2002). In order to assess the role of telomerase during the aging process, we used *tert*^−/−^, *tert*^+/+^ and *tert*^+/−^ specimens from the same genetic background and they were maintained in the same laboratory conditions. We examined fish survival during 110 weeks and obtained Kaplan-Meier survival curves for the three genotypes and found that *tert*^−/−^ zebrafish showed a reduced median lifespan (67.57 weeks) compared with *tert*^+/−^ (105.43 weeks) and *tert*^+/+^ (>110 weeks) (Fig. 2A).

Older zebrafish show spinal curvature due to muscle degeneration, and various degrees of curvature can be observed (Gerhard and Cheng, 2002; Gerhard et al., 2002). We noted that, whereas the *tert*^+/+^ zebrafish did not show spinal curvature before 24 months, *tert*^−/−^ and *tert*^+/−^ zebrafish started to show this sign of aging from 5 and 11 months of age, respectively (Fig. 2B). Moreover, 10% of *tert*^−/−^ zebrafish from 3- to 12-months old manifested spinal curvature compared with 1.2% of *tert*^+/−^ zebrafish and 0% of *tert*^+/+^ fish. Histopathological examination of liver sections from *tert*^+/+^ and *tert^+/−^* genotypes stained with H&E or PAS revealed no obvious microscopic alterations (Fig. 2C). In contrast, liver sections from *tert*^−/−^ specimens showed cytoplasmic vacuolization of hepatocytes coinciding with PAS-positive areas (Fig. 2C), suggesting the cytosolic accumulation of lipofuscin, an aging biomarker (Kishi et al., 2009), in hepatocytes. Similarly, histopathological examination of the retina from *tert*^+/+^ and *tert*^+/−^ zebrafish did not reveal any microscopic alterations (Fig. 2C), whereas retina from *tert*^−/−^ zebrafish showed a clear atrophy of the layer of rod and cones, which had a disorganized appearance, and a reduction in the thickness of the pigment epithelium, suggesting retinal cell degeneration (Fig. 2C).

**Fig. 3.**
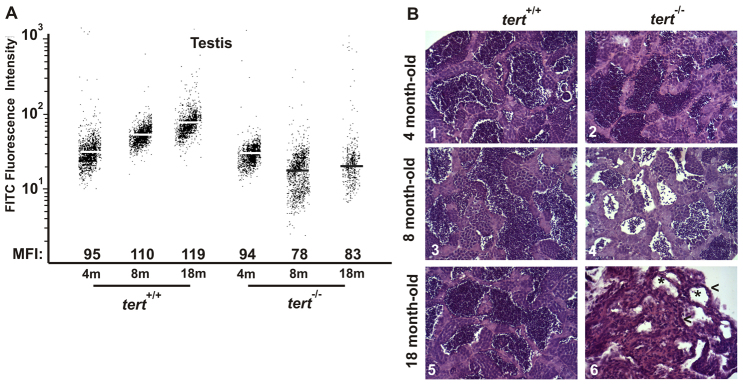
**Premature alterations of the testis tissue of *tert* mutant zebrafish.** (A) Representation of testis cell distribution from the wild-type and *tert* mutant genotype according to their telomere length. Medium fluorescence intensity (MFI) is indicated for each genetic background. The same trend was observed in the three independent experiments. (B) Representative image of zebrafish testis (*n*=3) from *tert^+/+^* and *tert^−/−^* backgrounds at 4, 8 and 18 months old (200×). Asterisks in panel 6 indicate atrophic tubules, and arrowheads atrophic stroma.

**Fig. 4.**
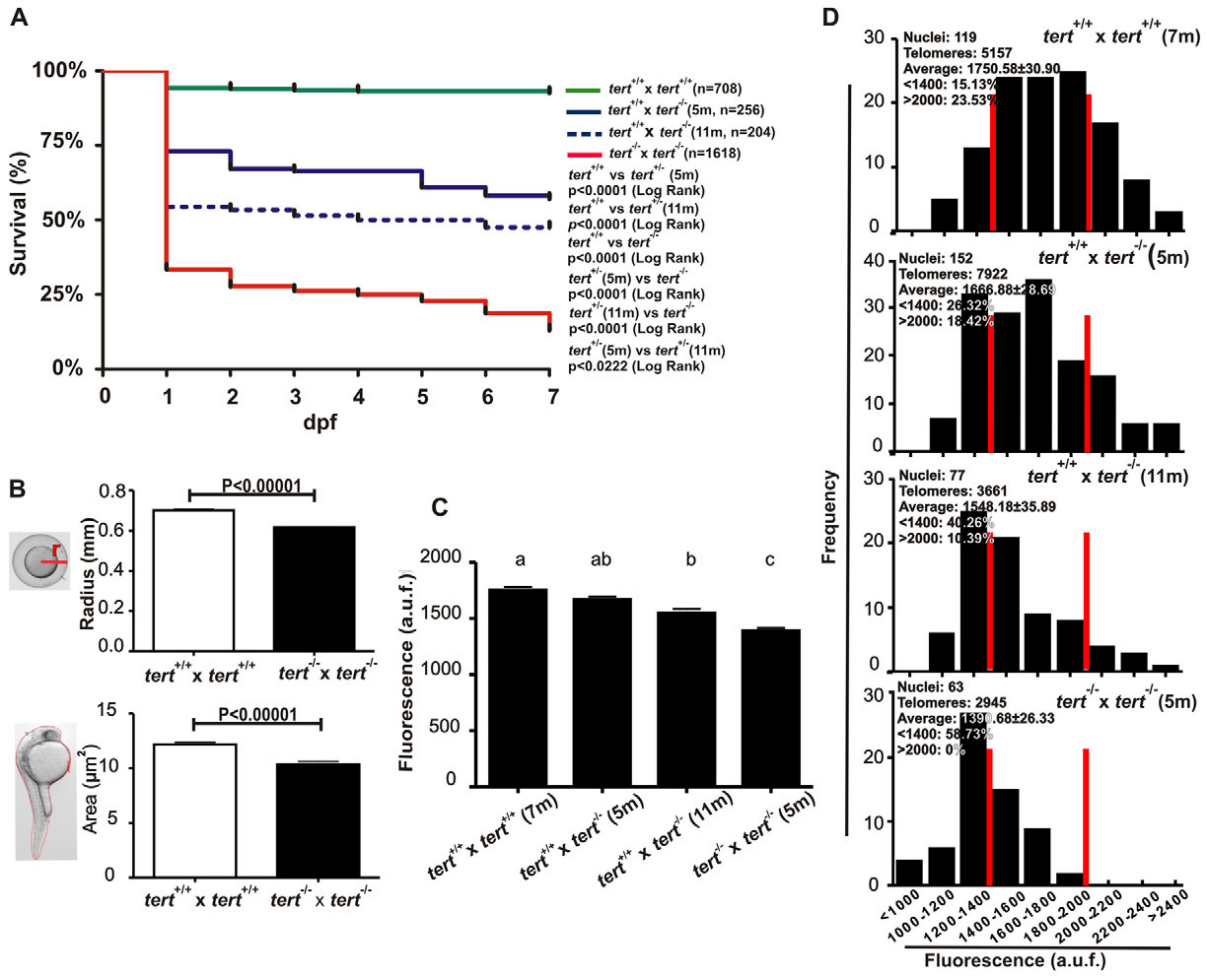
***tert* mutant zebrafish can be bred for only one generation.** (A) Larval survival curve (Kaplan-Meier representation) of four different genetic backgrounds. *n*, number of zebrafish per genotype. Statistical significance was assessed using the Log Rank test. (B) *tert* mutant zebrafish eggs and their corresponding dechorionated embryos had a smaller size than their wild-type siblings at 24 hpf (*n*≥50). Statistical significance according to Student’s *t*-test (*P*<0.00001). (C) Mean telomeric fluorescence values of 24-hpf embryos (*n*≥100) from wild-type and mutant genetic backgrounds. Data are mean values ± s.e.m. and different letters (a, b, c) denote statistically significant differences among telomeric fluorescence values of each sample according to a Tukey test (*P*<0.05). (D) Histograms showing telomere fluorescence frequencies. The red lines demarcate the percentage of the shortest (<1400 a.u.f.) and the longest (>2000 a.u.f.) telomeres. a.u.f., arbitrary units of fluorescence.

**Fig. 5.**
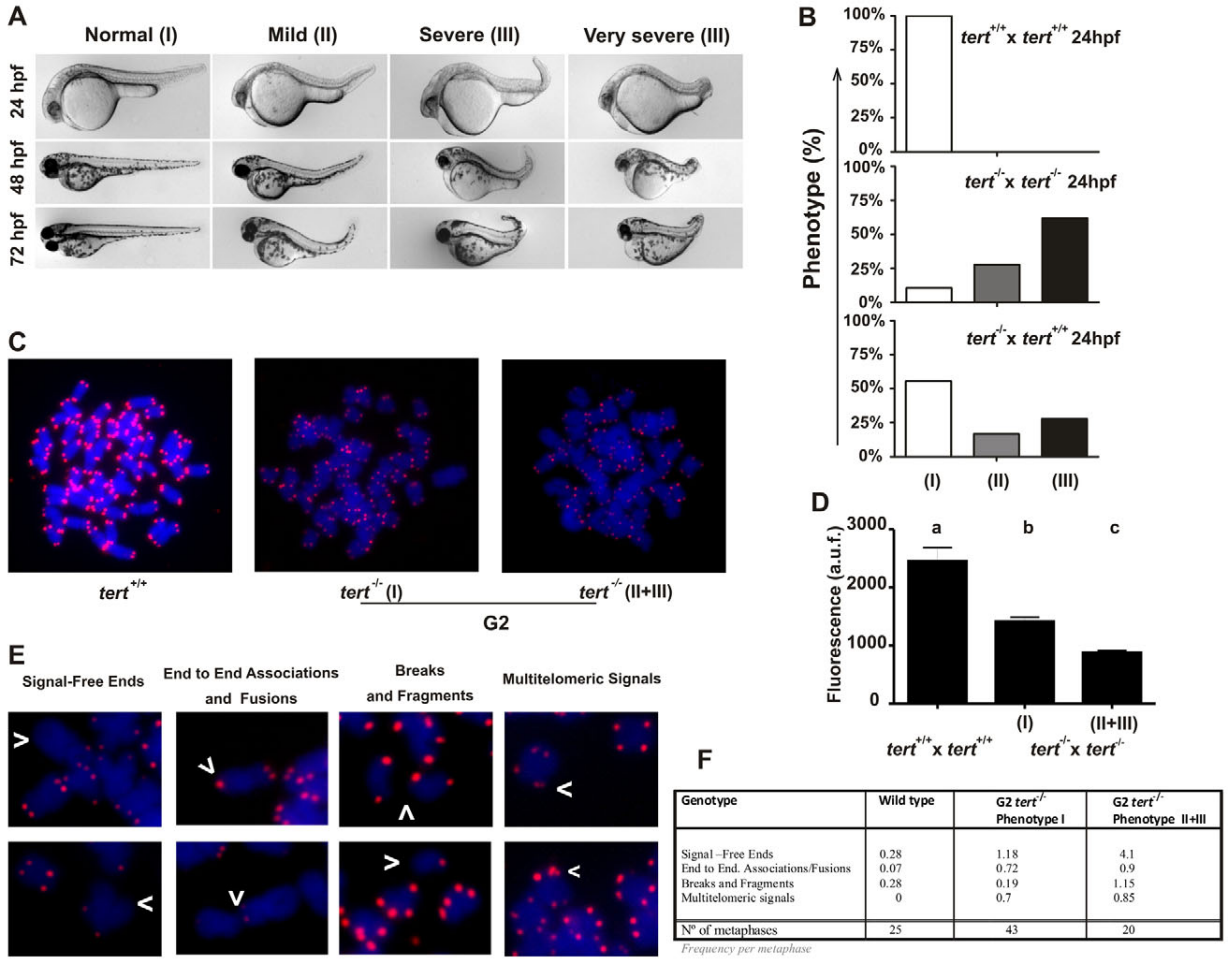
**Telomere dysfunction and chromosomal aberrations in G2*tert* mutant zebrafish.** (A) Representative image of the three groups of larvae resulting from *tert*^−/−^ × *tert*^−/−^ and *tert*^+/+^ × *tert*^−/−^ parental matings. The phenotypes were scored as wild type (I), mildly affected (II) and severely affected (III), as indicated in the Materials and Methods. (B) Percentage of larval survival of the three groups observed in A at 1 dpf. (C) Metaphase chromosomes from *tert^+/+^* and *tert^−/−^* cells were stained with DAPI, and the telomeres were visualized using FISH analysis. The phenotypes of mutant larvae were scored as indicated above. (D) Mean telomeric fluorescence values of 24-hpf embryos (*n*≥100) from wild-type and mutant genetic backgrounds. Data are mean values ± s.e.m. Different letters (a, b, c) denote statistically significant differences among telomeric fluorescence values of each sample according to a Tukey test (*P*<0.05). (E) Examples of chromosomal aberrations. (F) Frequency of chromosomal aberrations in cell from 24-hour larvae. a.u.f., arbitrary units of fluorescence.

**Fig. 6.**
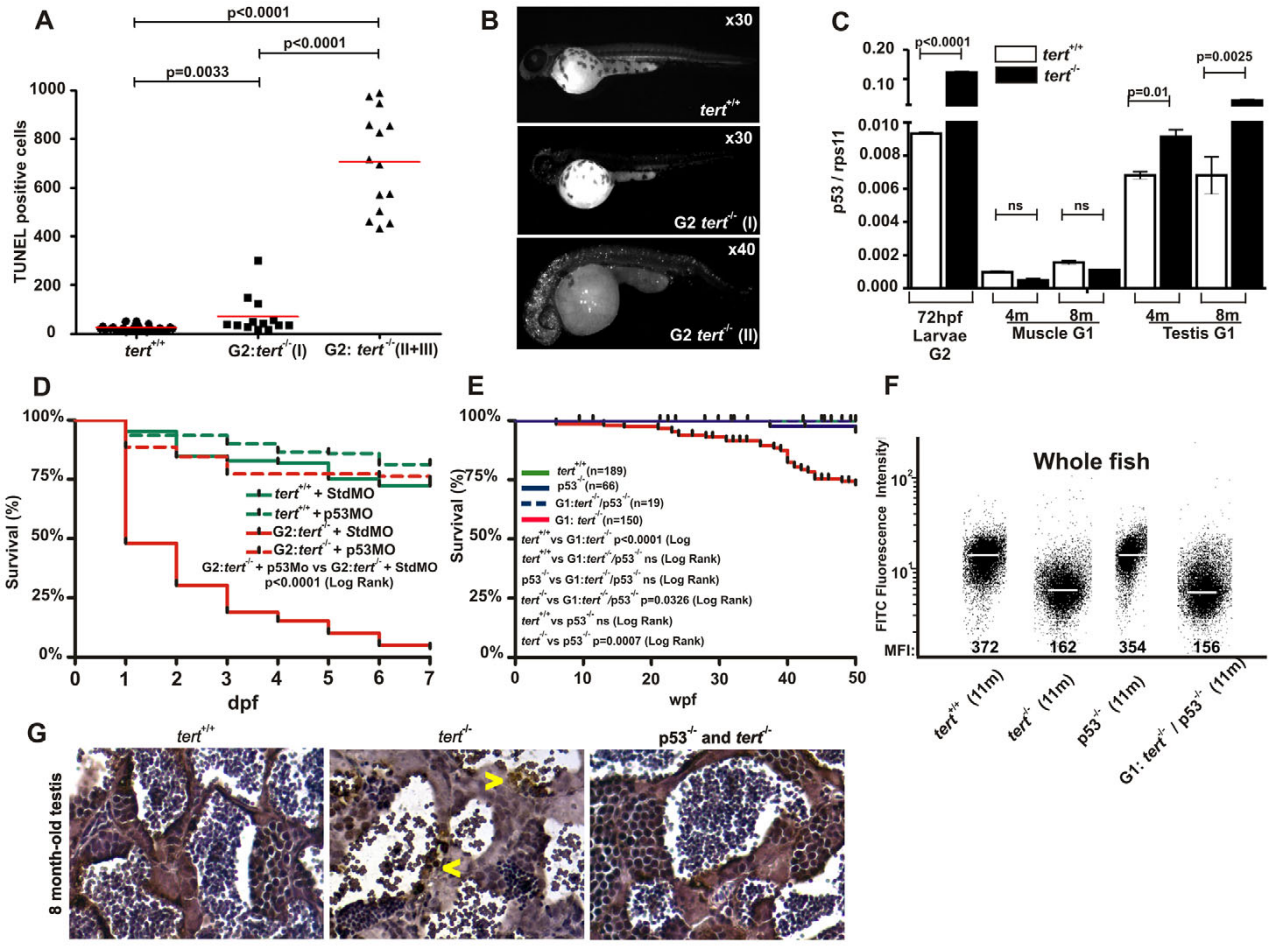
**p53 is responsible for the induction of apoptosis, developmental malformations and mortality of G1 and G2*tert* mutant zebrafish.** (A) Quantification of apoptotic cells based on TUNEL staining (see Materials and Methods). Significance was determined based on Student’s *t*-test (*P*<0.005). (B) Examples of embryos after TUNEL staining. (C) The mRNA levels of the *p53* gene were determined by real-time RT-PCR in 72-hpf larvae, and muscle and testis tissues of adult zebrafish, in the indicated genotypes. Gene expression is normalized against *rps11*. Each bar represents the mean ± s.e.m. from 100 pooled animals for larvae and three individual fish for adult tissue and triplicate samples. (D) Survival of G2 *tert*^−/−^ larvae microinjected at the one-cell stage with a morpholino against *p53*. (E) Kaplan-Meier representation of the survival of four genetic backgrounds. Post-hatching time is shown in weeks. The Log Rank test was used for statistical analysis. (F) Representation of total cell distribution from the four genotypes at the same age (11 months old) according to their telomere length. Medium fluorescence intensity (MFI) is indicated for each genetic background. The same trend was observed in the three independent experiments. (G) TUNEL assay in zebrafish testis sections from 8-month-old wild-type, *tert* mutant and double-mutant *tert*^−/−^*; p53*^−/−^ zebrafish (400×). Arrowheads indicate apoptotic cells.

By collecting data for 2 years, we observed a premature infertility in *tert*^−/−^ zebrafish and, therefore, we investigated possible differences in the reproductive capacity of the *tert* mutant zebrafish. With regards to clutch size and viability, the three genotypes showed a similar behavior in the 4- to 10-month-old period, but after that *tert*^−/−^ female zebrafish showed a reduction in the number of eggs per spawn, precisely when *tert*^+/+^ and *tert*^+/−^ females increased their reproductive performance. A flow-FISH assay performed on testis cells from *tert*^+/+^ zebrafish showed a net constant increase of their telomere length throughout their lifespan, whereas the telomere length of testis cells from *tert*^−/−^ zebrafish suffered an abrupt decline at the young-adult stage (8-months old) (Fig. 3A), which might be associated with premature infertility. The histological analysis of telomerase-deficient testes and their wild-type siblings at three different stages further confirmed this result. Both 4-month-old specimens and testis tissue sections from *tert^+/+^* (Fig. 3B, 1) and *tert^−/−^* (Fig. 3B, 2) zebrafish showed similar microscopic morphology, with the structure of seminiferous tubules and apparently normal connective stroma. At an age of 8 months, *tert^+/+^* presented the same morphology as at 4 months of age (Fig. 3B, 3), whereas atrophic seminiferous tubules in *tert^−/−^* testis tissue were apparent (Fig. 3B, 4). Finally, at 18 months, whereas the *tert^+/+^* zebrafish preserved the normal testicular architecture (Fig. 3B, 5), a complete atrophy of both tubules and stroma and the absence of spermatozoa were observed in the testes of *tert^−/−^* zebrafish (Fig. 3B, 6). TUNEL assay confirmed the presence of apoptotic cells in the seminiferous tubules of telomerase-deficient testes, which was more evident at 8 months of age (see below). These results correlate well with the decrease in telomere length and suggest a direct link between telomerase function, fertility and aging.

### Telomerase-deficient zebrafish can be bred for only one generation

The deletion of either mouse telomerase component, TERT or TR, did not show any aberrant phenotype in the first few generations, when telomeres were long (Chiang et al., 2004; Lee et al., 1998). A similar phenotypic delay in the response to telomerase loss has been described in *C. elegans* and *A. thaliana* TERT mutants, again with the first generation of telomerase-null mutants being phenotypically normal (Cheung et al., 2006; Fitzgerald et al., 1999). However, all these models develop abnormalities and chromosomal instability after several generations.

We wanted to check whether successive generations would show a more apparent phenotype. At 1 day post-fertilization (dpf), only 32% of second-generation (G2) telomerase-deficient zebrafish larvae obtained from *tert*^−/−^ parental matings survived, compared with 95% survival of wild-type larvae (Fig. 4A). Curiously, we observed that *tert* mutant zebrafish eggs and their corresponding dechorionated embryos were smaller than *tert^+/+^* zebrafish eggs and 24-hour-post-fertilization (hpf) embryos (Fig. 4B), as found in late generations of telomerase-deficient mice, which have fewer functional stem cells within tissues (Flores and Blasco, 2009; Flores et al., 2005). Importantly, the larvae obtained from *tert*^+/+^ × *tert*^−/−^ parental mating showed increased survival at 1 dpf, indicating that the reintroduction of one wild-type *tert* allele was able to rescue the viability of the offspring. However, the recovery was not complete, which suggested haploinsufficiency of the *tert* gene. We also observed that the survival of larvae obtained by breeding young (5-month-old) *tert^−/−^* mutant males with *tert^+/+^* females was significantly higher than when the males were older (11-months old) (73% versus 54% survival, respectively) (Fig. 4A).

Telomerase-deficient mice show telomere shortening in successive generations (Blasco et al., 1997). Therefore, we wanted to determine the telomere length of G2 *tert*^−/−^ progeny. We used Q-FISH in interphasic nuclei to measure the mean telomere length of 24-hpf larvae. Compared with the *tert*^+/+^ progeny (1750.58±30.90 a.u.f.), larvae obtained from *tert*^−/−^ parental matings showed a significant telomere shortening (1390.68±26.33 a.u.f.) (Fig. 4C). The telomere-length frequency histograms, where the percentage of very short (<1400 a.u.f.) and very long (>2000 a.u.f.) telomeres is shown, reveal that the G2 *tert*^−/−^ progeny had a higher percentage (58.73%) of very short telomeres compared with the *tert*^+/+^ genotype (15.13%) (Fig. 4D).

Because we observed a better survival rate in the offspring obtained from 5- rather than 11-month-old *tert^−/−^* mutant males outcrossed with *tert*^+/+^ females, we examined whether the telomere length was also rescued in both progenies. It was found that telomere length was shorter for the progeny of the older telomerase-deficient males (1548.18±35.89 a.u.f. versus 1666.88±28.69 a.u.f.) (Fig. 4C). In addition, the telomere length frequency histogram revealed 40.26% of very short telomeres for the progeny of 11-month-old male progenitors versus 26.32% in the case of 5-month-old males (Fig. 4D).

We next studied the gross morphology of 24- to 72-hpf embryos obtained from *tert*^−/−^ × *tert*^−/−^ and *tert*^+/+^ × *tert*^−/−^ parental matings and observed three different phenotypes (groups I–III), whereas embryos obtained from *tert*^+/+^ × *tert*^+/+^ and *tert*^+/−^ × *tert*^+/−^ parental matings showed a normal phenotype and development (Fig. 5A). Embryos belonging to group I showed an apparently normal phenotype; embryos in group II had mild defects, such as a bent caudal fin; and embryos from group III showed serious developmental defects, such as a bent caudal fin, pericardial edema and general malformations. We also found that the G2 *tert*^+/−^ progeny (obtained from *tert*^+/+^ × *tert*^−/−^ parental matings) had a high percentage of larvae in group I, whereas the G2 *tert*^−/−^ progeny had a high percentage of larvae belonging to group III, as well as a higher mortality rate (Fig. 5B). Collectively, these data further corroborate the rescue of offspring viability by the restoration of one wild-type *tert* allele.

We next investigated whether telomere length directly correlated with the larval phenotype. To do this, we performed a Q-FISH assay in metaphasic nuclei to compare the telomere length of group I G2 *tert*^−/−^ progeny with that of the G2 progeny showing an abnormal phenotype (groups II and III) at 48 hpf. Besides the expected difference between the mean telomere length of cells from *tert*^+/+^ and G2 *tert*^−/−^ embryos, we observed that G2 embryos with an abnormal phenotype (groups II and III) had significantly shorter mean telomere length than their normal counterparts (group I) (Fig. 5C,D). Finally, we carried out a detailed analysis of the chromosomal defects of cells from both the normal and abnormal phenotypes (Fig. 5F) and observed an increase in telomere-free chromosome ends, chromosome breakages and chromosomal fragments in metaphasic nuclei from embryos with an abnormal phenotype (Fig. 5E). Interestingly, G2 *tert^−/−^* embryos also showed an increased proportion of chromosome ends with multiple telomeric signals (MTSs), a type of aberration related to increased telomere fragility (Martínez et al., 2009; Sfeir et al., 2009). These chromosomal aberrations could explain the phenotypic differences observed between G2 *tert*^−/−^ normal embryos and those showing severe developmental defects and malformations.

### Critically short telomeres activate p53

The high mortality of *tert*^−/−^ G2 progeny drove us to perform a whole-mount TUNEL assay to detect and quantify the presence of apoptotic cells. At 48 hpf, *tert*^−/−^ embryos showed a significantly higher number of apoptotic cells than did wild-type embryos. More importantly, *tert*-deficient embryos with an abnormal phenotype (groups II and III) had a much higher number of apoptotic cells than did mutant embryos with a normal phenotype (group I) (Fig. 6A,B).

To test whether the high percentage of apoptotic cells detected in *tert*^−/−^ G2 progeny could be the result of p53 activation through a DNA damage signal, such as telomere dysfunction (Maser and DePinho, 2004), we analyzed *p53* gene expression using real-time reverse transcriptase PCR (RT-PCR) and found that the mRNA levels of p53 were significantly higher in G2 *tert*^−/−^ larvae than in their *tert*^+/+^ siblings at 72 hpf (Fig. 6C). Notably, the transcript levels of *p53* were also slightly higher in the testes of 4-month-old G1 *tert*-deficient adults and became especially evident, and statistically significant, in 8-month-old adults, coinciding with the onset of testicular atrophy (Fig. 3B). These results, together with the decrease in telomere length of testicular cells at the same age (Fig. 3A), confirm the crucial importance of telomerase in this organ.

To ascertain whether p53 activation was responsible for the induction of apoptosis, developmental malformations and mortality of G2 *tert^−/−^* larvae, we inhibited p53 expression using a specific morpholino. The results showed that genetic depletion of p53 results in a completely normal development and survival of G2 *tert*^−/−^ embryos (Fig. 6D). Furthermore, we assessed the effect of the permanent absence of p53 in *tert*-deficient fish by obtaining the G1 of double-mutant *tert^−/−^; p53^−/−^* zebrafish. To this end, we obtained survival curves covering 50 weeks and used *tert^−/−^* and *p53^−/−^* single-mutant zebrafish lines as a reference for normal longevity. Although the G1 *tert*^−/−^ zebrafish had reduced longevity (*P*=0.0326), the survival of the G1 *tert*^−/−^*; p53*^−/−^ line showed no significant differences with those of their *tert^+/+^* or *p53^−/−^* siblings (Fig. 6E). Notably, G1 *tert*^−/−^ cells had a similar mean telomere signal than their G1 *tert*^−/−^*; p53*^−/−^ siblings (Fig. 6F). Furthermore, the histopathological analysis of the male zebrafish testes revealed a normal morphology and the absence of TUNEL^+^ germ cells (Fig. 6G), suggesting that p53 senses telomere damage in germ stem/progenitor cell populations leading to massive germ cell apoptosis, and that p53 deficiency did not influence telomere length in *tert*-deficient zebrafish, which is the same as what occurs in mice (Chin et al., 1999).

**Fig. 7.**
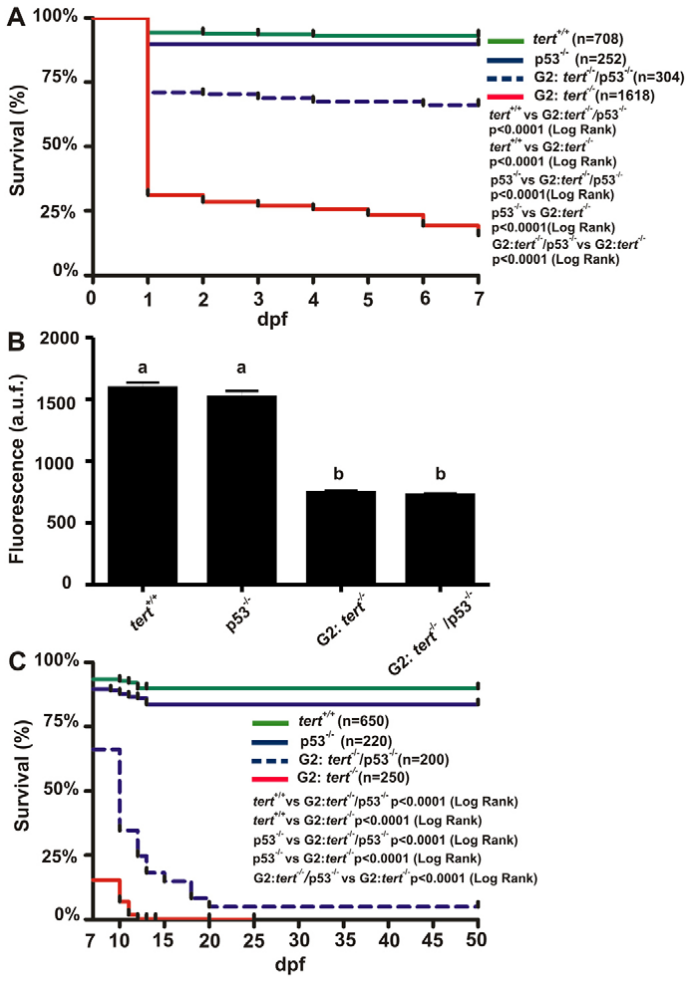
**Survival and telomere length of G2*tert*^−/−^ zebrafish in a *p53*^−/−^ background.** (A) Larval survival curve (Kaplan-Meier representation) of four different genetic backgrounds. *n*, number of zebrafish per genotype. Statistical significance was assessed using the Log Rank test. (B) Mean telomeric fluorescence values of 24-hpf embryos (*n*≥100) from wild-type and mutant genetic backgrounds. Data are mean values ± s.e.m. and different letters (a, b) denote statistically significant differences between telomeric fluorescence values of each sample according to a Tukey test (*P*<0.05). (C) Kaplan-Meier representation of the survival of four genetic backgrounds. a.u.f., arbitrary units of fluorescence.

Because the knockdown of p53 with morpholino was transient (Fig. 6D), we obtained the G2 *tert^−/−^; p53^−/−^* line to examine whether p53 deficiency was able to rescue the longevity of the G2 *tert^−/−^* line. Fig. 7A shows that p53 deficiency partially rescued the viability of the G2 *tert^−/−^* zebrafish in the first week (*P*<0.0001). However, the telomere length was unaffected (Fig. 7B). Notably, although we observed a high percentage of mortality in the G2 *tert^−/−^; p53^−/−^* line between 10 and 20 dpf, there were statistically significant differences between the survival of singly and doubly null mutants (Fig. 7C). Together, these data indicate that p53 is able to rescue the survival of *tert*-deficient fish but not telomere length.

## DISCUSSION

The study of telomere and telomerase biology is crucial to the understanding of aging and cancer processes. Although the mouse has been extensively used as a model for these purposes and has been established as a key model to elucidate the role of telomeres and telomerase in aging and cancer, there are fundamental differences between mice and humans. For example, mouse telomere length is much longer than that of humans and the mouse model is not able to completely recapitulate the symptoms of human telomerase deficiency (Autexier, 2008; Mitchell et al., 1999). Therefore, we have characterized a second vertebrate model for studying the role of telomerase and telomeres in aging research. Zebrafish telomeres (15–20 kb) are relatively similar to human ones (10–15 kb) and show a progressive shortening (Anchelin et al., 2011). The telomerase mutant zebrafish line used in this study was obtained from the Sanger Institute (hu3430 line). This mutation leads to a premature stop codon resulting in a truncated protein removing the RNA-binding and reverse transcriptase domains. As expected, this mutant lacks telomerase activity and shows shorter telomeres than wild-type fish. However, we observed an increased expression of *tert* mRNA, suggesting that the absence of telomerase activity induces the activation of its own promoter to try to compensate for this. This result indicates the existence of regulatory mechanisms of *tert* gene expression that would be worth exploring in future studies.

An increased telomere length from larvae to adult fish stages followed by telomere shortening in aged fish has previously been reported (Anchelin et al., 2011). These studies have been performed using whole zebrafish specimens from various genetic strains. Unexpectedly, in the present study, the telomeric length in *tert* mutant fish, although shorter than that of the wild type, remained constant or even increased slightly during aging. However, the dynamics of telomere length varied during the zebrafish life cycle in different organs and seems to be tissue specific. Although there was a tendency to a slight decrease in telomere length in the muscle, the decrease in the testis was much more pronounced. In sharp contrast, kidney cells maintained their telomere length throughout life. Although these results might be explained by differences in cell proliferation rates in each tissue (Lee et al., 1998), it is tempting to speculate about the involvement of the activation of telomerase-independent telomere maintenance mechanisms [ALT (alternative lengthening of telomeres)], at least in the highly proliferative hematopoietic cells of the kidney. Therefore, the *tert*-deficient zebrafish model described in this study might be an excellent model for investigating the role of ALT in telomere maintenance in the absence of telomerase activity.

Telomerase-null mice have no discernible phenotypes in the first generations (G1–G5) because mice have very long telomeres (Blasco et al., 1997; Liu et al., 2000). Only when the telomeres become critically short in later generations (G5 and G6) do telomerase-deficient mice show prominent premature aging symptoms, including impaired spermatogenesis and loss of fertility, bone marrow failure, atrophy of the small intestine, and immunosenescence-related disease (Herrera et al., 1999; Lee et al., 1998). However, because humans and zebrafish have a similar telomere length, we were able to observe premature aging symptoms in the G1 of *tert*-deficient zebrafish, with the most apparent phenotype being the sharp decline in the mean life expectancy of *tert* mutant zebrafish. Interestingly, *tert*^+/−^ zebrafish seemed to have a reduced longevity compared with *tert*^+/+^. Several signs of aging already described in the zebrafish (Gerhard and Cheng, 2002; Gerhard et al., 2002; Kishi et al., 2009) were prematurely observed in young (10-month-old) *tert* knockout mutants, such as a higher percentage of *tert^−/−^* individuals exhibiting extreme thinness and/or spinal curvature compared with *tert*^+/−^ and *tert^+/+^* siblings (10% versus 1.2% and 0%, respectively), increased lipofucsine accumulation in the liver, and retinal cell degeneration. By contrast, telomere shortening and genome instability in late-generation telomerase-deficient mice, as well as in humans, is associated with germ cell depletion in the testis (Hemann et al., 2001; Lee et al., 1998). Similarly, we also observed the dramatic consequences of telomere shortening in premature testis degeneration and infertility.

In order to study the phenomena of aging earlier in time, we created a G2 of the *tert* mutant and found that almost all G2 embryos/larvae died before the first week of age. These larvae exhibited shorter telomeres (58.73% versus 15.13% in wild type) and were smaller than wild-type larvae, as happens in a percentage of late-generation mouse *TR^−/−^* embryos owing to the effect of short telomeres on stem cell functionality (Flores et al., 2005; Herrera et al., 1999). Interestingly, the reintroduction of the *tert* gene in *tert*-deficient zebrafish with inherited short telomeres prevented further telomere shortening and organism mortality, as also occurs in mice (Bernardes de Jesus et al., 2012; Jaskelioff et al., 2011; Samper et al., 2001). This result indicates that a minimum telomere length is necessary to maintain normal tissue homeostasis and, more importantly, that there are therapeutic benefits of new drugs that are able to induce the expression of the wild-type *TERT* or *TR* allele in heterozygous DC patients. The powerful advantages of the zebrafish for high-throughput drug screening (Zon and Peterson, 2005) could contribute to the identification of such drugs.

It is noteworthy that the telomere length of the offspring obtained from wild-type females and 11-month-old *tert^−/−^* males was shorter than that of the offspring of wild-type females and 5-month-old *tert^−/−^* males. Moreover, this correlated with the better survival of the offspring from younger mutant males and, therefore, is indicative of an anticipation phenomenon related with telomere length, which has been described in individuals with DC (Armanios et al., 2005; Vulliamy et al., 2004). This anticipation phenomenon could be explained by a haploinsufficiency of the *tert* gene, because the partial telomere length rescue of *tert* heterozygotes was reflected in better organism survival. However, these heterozygotes still had a high proportion of short telomeres compared with the wild type, indicating that telomerase dosage was crucial. Therefore, the *tert*-deficient zebrafish line recapitulates the mechanisms of age in telomerase-deficient mice and individuals with DC (Armanios et al., 2005; Knudson et al., 2005; Vulliamy et al., 2004).

A very recent study has shown that the rate of increase in the percentage of short telomeres, rather than the rate of telomere shortening per month, was a significant predictor of lifespan in both wild-type and telomerase-deficient mice, and those individuals who showed a higher rate of increase in the percentage of short telomeres were also the ones with a shorter lifespan (Vera et al., 2012). Consistent with this idea, the G1 *tert*-deficient zebrafish showed a shorter lifespan than the wild type, and the G2 died prematurely. In addition, the presence of several phenotypes in the G2 of *tert*-deficient zebrafish further supports this notion, because the degree of developmental defects of each phenotype was directly correlated with telomere length and, specifically, with the proportion of short telomeres. Notably, telomere shortening was also accompanied by an increase in the number of signal-free chromosome ends, chromosome fusions and MTSs. Therefore, we speculate that *tert* heterozygous zebrafish with a higher proportion of short telomeres would show an anticipation in aging signals and a shorter longevity. However, further studies are required to characterize *tert* heterozygote zebrafish and to study whether they are able to model the anticipation process observed in individuals with DC.

The pathologies that occur in the telomerase-deficient mouse model are accompanied by a reduction in the proliferative potential due to an activation of p53, which leads to growth arrest and/or apoptosis in the affected tissues (Leri et al., 2003). In addition, p53 deficiency rescues the adverse effects of telomere loss (Chin et al., 1999; Flores and Blasco, 2009). These results are consistent with the induction of *p53* gene expression in zebrafish tissues with a high proliferation rate, such as those of the testis. In this tissue, *p53* gene expression was higher in 8-month-old *tert* mutant zebrafish compared with their wild-type siblings, coinciding with telomere shortening. Similarly, G2 larvae also showed an activation of *p53* gene expression and an increased number of apoptotic cells in the abnormal phenotypes (groups II and III) compared with the normal one (group I), indicating once again the importance of critically short telomeres and genomic instability. The relevance of p53 in the premature senescence and reduced fertility of G1 and the developmental defects and early mortality of G2 *tert*-deficient larvae was confirmed by the complete rescue of G2 larval survival by transient p53 inactivation and the generation of G1 *tert^−/−^; p53^−/−^* double mutants, which showed an absence of morphological alterations and TUNEL^+^ germ cells in the testes at 8 months of age. Importantly, the G2 *tert^−/−^; p53^−/−^* animals had increased longevity, despite the fact that their telomere length did not increase. These results might explain the high percentage of mortality between 10 and 20 dpf of G2 doubly null mutants. Although p53 deficiency was able to initially rescue the adverse effects of telomere loss, sustained cell proliferation in the absence of p53 might result in a progressive telomere shortening and dysfunction that, in turn, would reduce lifespan. Further aging and carcinogenesis studies are required with the G2 doubly null mutants.

To summarize, the telomerase-deficient zebrafish characterized in this study should be considered as a promising model to study telomere-driven aging, because they are able to recapitulate human telomere and telomerase biology. In addition, it is an exceptional vertebrate model for the discovery of new treatments able to temporarily reactivate telomerase expression in individuals with DC.

## MATERIALS AND METHODS

### Maintenance of zebrafish

Wild-type AB zebrafish (*Danio rerio*) were obtained from the Zebrafish International Resource Center (ZIRC). The *tert* mutant line (allele hu3430) was obtained from the Sanger Institute and the *p53* mutant line *zdf1* (P53^M214K^) (Berghmans et al., 2005) was kindly provided by Leonard I. Zon (HSCRB, Harvard University, Cambridge, MA). Zebrafish were maintained in recirculating tanks following instructions from ‘*The zebrafish book*’ (Westerfield, 2000). Adult fish were maintained at 26°C, with a 14:10 hour light:dark cycle and were fed twice daily, once with dry flake food (PRODAC) and once with live artemia (MC 450, INVE AQUACULTURE). Zebrafish embryos were maintained in egg water at 28.5°C and were fed at 5 days with NOVOTOM and with live artemia at 11 days of life.

The experiments performed comply with the Guidelines of the European Union Council (86/609/EU) and were approved by the Bioethical Committee of the University Hospital Virgen de la Arrixaca (Spain) under approval number PI06/FIS0369/040706.

### Cell isolation

Zebrafish were anesthetized at different stages with 0.05% benzocaine, briefly rinsed in 0.5% chilled bleach, crushed and incubated in phosphate buffered saline (PBS) supplemented with antibiotics for 30 minutes, centrifuged (600 ***g***, 5 minutes), incubated in trypsin (0.5 mg/ml)/EDTA (0.1 mg/ml) in PBS for 1 minute, centrifuged (600 ***g***, 5 minutes) and then incubated in collagenase (0.5 mg/ml) in RPMI medium supplemented with CaCl_2_ 2H_2_O (0.7 mg/ml) for 30 minutes. The cell suspensions were obtained by pipetting, smashing and finally filtering the digested tissues through a 100 μm mesh, washed and resuspended in PBS.

### Telomerase activity assay

A real-time quantitative TRAP (Q-TRAP) analysis was performed as described by Herbert et al. (Herbert et al., 2006). The protein extracts were obtained as described by the authors. To quantify the telomerase activity, PCR amplification was performed as indicated by the authors. We performed the standard curve as described by Anchelin et al. (Anchelin et al., 2011). After PCR, real-time data were collected and converted into relative telomerase activity (RTA) units performing the calculation: RTA of sample=10 (Ct sample-γint)/slope. The standard curve obtained was: *y*=–3.2295x+23.802.

### qPCR analysis

Total RNA was extracted from whole zebrafish larvae and different zebrafish tissues at several ages using the TRIzol Reagent (Invitrogen), following the manufacturer’s instructions, and was treated with DNase I Amplification grade (1 unit/μg RNA, Invitrogen). The SuperScript III RNase H-Reverse Transcriptase (Invitrogen) was used to synthesize first-strand cDNA with oligo-dT18 primer from 1 μg of total RNA at 50°C for 60 minutes.

Real-time PCR was performed with an ABI PRISM 7700 instrument (Applied Biosystems) using SYBR Green PCR Core Reagents (Applied Biosystems). Reaction mixtures were incubated for 10 minutes at 95°C, followed by 40 cycles for 15 seconds at 95°C, for 1 minute at 60°C, and finally for 15 seconds at 95°C, for 1 minute at 60°C, and 15 seconds at 95°C. For each mRNA, gene expression was corrected by the ribosomal protein S11 (*rps11*) content in each sample. The primers used were TERT-F2: 5′-CGGTATGACGGCCTATCACT-3′ and TERT-R1: 5′-TAAACGGCCTCCACAGAGTT-3′ for zebrafish *tert*, P53-F: 5′-GATAGCCTAGTGCGAGCACAC-3′ and P53-RWT: 5′-AGCTGCATGGGGGGGAT-3′ for zebrafish *p53*, and F: 5′-ACAGAAATGCCCCTTCACTG-3′ and R: 5′-GCCTCTTCTCAAAACGGTTG-3′ for *rps11*.

### Histological analysis

Several adult tissue samples (eye, liver and testicle) were fixed in 4% buffered formalin (Panreac Química) for 24 hours, processed and paraffin-embedded. Histological hematoxylin-eosin (H&E) and periodic acid Schiff’s (PAS) staining were carried out in 4-μm sections using standard protocols. Stained histological sections were examined using conventional light microscopy at 200×, 400× and 630×.

TUNEL assay was carried out on zebrafish testis tissue from *tert^+/+^* and *tert^−/−^*, using a DeadEnd^TM^ Colorimetric Apoptosis Detection System (Promega) following the manufacturer’s instructions. Stained histological sections were examined by conventional light microscopy at 400×.

### Metaphase spreads

Methods were adapted from Lee and Smith (Lee and Smith, 2004). At 23 hpf, zebrafish embryos previously dechorionated by pronase treatment were treated with colchicine (4 mg/ml) for 8 hours. Ice-cold 0.9× PBS with 10% fetal bovine serum was added and embryos filtered subsequently through a 100 μm and 40 μm mesh. Cell suspensions were then centrifuged at 250 ***g*** for 10 minutes at 4°C. The supernatant was decanted and cells were incubated for 25 minutes at 28.5°C in a hypotonic solution (1.1% sodium citrate, 4 mg/ml colchicine). Cells were centrifuged at 450 ***g*** for 10 minutes at 4°C and ice-cold Carnoy’s methanol: glacial acetic acid fixative (3:1, v:v) was added. This last step was repeated, and chromosomes were then dropped onto microscope slides and allowed to dry overnight at 37°C.

### Q-FISH

Q-FISH on interphasic and metaphasic cells was performed as described in Canela et al. (Canela et al., 2007). Cy3 and DAPI images were captured with 100× and 60× objectives, respectively, using a Nikon Digital Camera DXM 1200C on a Nikon Direct Eclipse fluorescence microscope. Telomere fluorescence signals were quantified using the TFL-TELO program (from Peter Lansdorp, Vancouver, Canada).

### Flow-FISH

One million cells from each sample was divided into two replicate tubes: one pellet was resuspended in a 500 μl hybridization buffer and another in a hybridization buffer without an FITC-labeled telomeric peptide nucleic acid (PNA) probe, as a negative control. Samples were then denatured for 10 minutes at 80°C under continuous shaking and hybridized for 2 hours in the dark at room temperature. After that, the cells were washed twice in a washing solution (70% deionized formamide, 10 mM Tris pH 7.2, 0.1% BSA, 0.1% Tween-20 in dH_2_O). The cells were then centrifuged at 600 ***g***, resuspended in 500 μl of propidium iodide solution, incubated for 2 hours at room temperature, stored at 4°C and analyzed by flow cytometry within the following 48 hours.

### Apoptosis assay in zebrafish

We performed a TUNEL assay on 48-hpf larvae from the *tert^+/+^* and *tert^−/−^* genotype, the latter being a normal phenotype (group I) and a defective phenotype (mild: group II, and severe and very severe: group III). We used the ApopTag Red *In Situ* Apoptosis detection kit (S7165-Millipore) following the manufacturer’s instructions. Apoptotic cells were counted using ImageJ software.

### Quantitative analysis and statistics

Data processing and statistical analyses were performed using Microsoft Excel and Graph Pad Prism version 5.01, which were used to generate each of the graphs shown in the figures, performing statistical tests where appropriate. Data were analyzed using ANOVA and a Tukey multiple range test to determine differences between groups. The differences between two samples were analyzed using Student’s *t*-test. The comparison of survival between mutant fish and their wild-type controls shown in the Kaplan-Meier survival curves was performed using the Log Rank test.
